# Vortices and antivortices in two-dimensional ultracold Fermi gases

**DOI:** 10.1038/srep45702

**Published:** 2017-04-04

**Authors:** G. Bighin, L. Salasnich

**Affiliations:** 1 IST Austria (Institute of Science and Technology Austria), Am Campus 1, 3400 Klosterneuburg, Austria; 2 Dipartimento di Fisica e Astronomia “Galileo Galilei” and CNISM, Università di Padova, Via Marzolo 8, 35131 Padova, Italy; 3 Istituto Nazionale di Ottica del Consiglio Nazionale delle Ricerche, Via Nello Carrara 1, 50019 Sesto Fiorentino, Italy

## Abstract

Vortices are commonly observed in the context of classical hydrodynamics: from whirlpools after stirring the coffee in a cup to a violent atmospheric phenomenon such as a tornado, all classical vortices are characterized by an arbitrary circulation value of the local velocity field. On the other hand the appearance of vortices with quantized circulation represents one of the fundamental signatures of macroscopic quantum phenomena. In two-dimensional superfluids quantized vortices play a key role in determining finite-temperature properties, as the superfluid phase and the normal state are separated by a vortex unbinding transition, the Berezinskii-Kosterlitz-Thouless transition. Very recent experiments with two-dimensional superfluid fermions motivate the present work: we present theoretical results based on the renormalization group showing that the universal jump of the superfluid density and the critical temperature crucially depend on the interaction strength, providing a strong benchmark for forthcoming investigations.

Quantized vortices are characterized by a circulation of the velocity field quantized in multiples of ħ/m^*^, where *ħ* is Planck’s constant and m^*^ is the mass of a superfluid particle, in the case of a bosonic superfluid, or the mass of a Cooper pair, in the case of a fermionic superfluid. Quantized vortices are a fundamental feature of superfluid and superconducting systems^1^ and have been observed in a wide variety of systems, including type-II superconductors^2^,^3^,^4^, superfluid liquid Helium^5^,^6^, superfluid liquid Helium nanodroplets^7^,^8^, ultracold gases^9^,^10^, and exciton-polaritons inside semiconductor microcavities^11^,^12^.

From a phenomenological standpoint quantized vortices resemble non-quantized vortices in classical hydrodynamical systems. The quantization of circulation is a peculiar consequence of the existence of an underlying *compact* real field, whose spatial gradient determines the local superfluid velocity of the system^13^,^14^. This compact real field, the so-called Nambu-Goldstone field, is the phase angle of the complex bosonic field which describes, in the case of attractive fermions, strongly-correlated Cooper pairs of fermions with opposite spins^14^.

In two-dimensional (2D) superfluid systems there can not be Bose-Einstein condensation and off-diagonal long-range order at finite temperature, as a consequence of the Mermin-Wagner-Hohenberg (MWH) theorem^15^,^16^,^17^. Nevertheless a vortex-driven phase transition at a finite temperature *T*_BKT_ is still present due to the Berezinskii-Kosterlitz-Thouless (BKT) mechanism^18^,^19^. Below the critical temperature *T*_BKT_ the system is superfluid and characterized by bound vortex-antivortex pairs and algebraic long-range order. Above *T*_BKT_, on the other hand, vortex-antivortex pairs unbind, free quantized vortices proliferate, and the system loses its superfluid properties with exponential decay of coherence. Within this scenario it is clear that quantized vortices play a key role in determining the finite-temperature properties of a 2D superfluid.

The rapid developments in the realization and manipulation of ultracold gases allow for the observation of dilute atomic vapors trapped in quasi-two-dimensional configurations. In 2006 the BKT transition and the associated unbinding of vortices has been observed in an atomic Bose gas by Hadzibabic *et al*.^9^; in this experiment, the proliferation of free vortices is directly imaged by letting two 2D clouds expand and interfere with each other; the free vortices can then be counted individually by looking at the number of defects in the interference pattern. The same transition was also observed by Schweikhard *et al*.^10^ in an optical lattice, using the usual absorption imaging technique of the vortex cores. Recent experiments^20^,^21^,^22^,^23^ deal with 2D attractive Fermi gases in the crossover from the weak-coupling BCS regime of largely overlapping Cooper pairs to the strong-coupling BEC regime of composite bosons and provide motivation for the present theoretical investigation.

## Results

### Single-particle and collective excitations in ultracold Fermi superfluids

In a fermionic superfluid with tunable *s*-wave interaction the mean-field theory predicts the existence of fermionic single-particle excitations, whose low-energy spectrum is


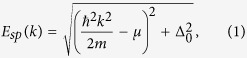


where *m* is the mass of a fermion, *μ* is the chemical potential of the system, and Δ_0_ is the pairing energy gap. The inclusion of beyond-mean-field effects, namely quantum fluctuations of the pairing field, gives rise to bosonic collective excitations^24^, whose low-energy spectrum across the BCS-BEC crossover is^25^,^26^


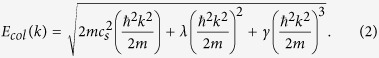


These collective excitations are density waves reducing to the Bogoliubov-Goldstone-Anderson mode *E*_*col*_(*k*) = *c*_*s*_*ħk* in the limit of small momenta. Here *c*_*s*_ is the speed of sound, while *λ* and *γ* are parameters taking into account the increase of kinetic energy due to the spatial variation of the density and depend on the strength of the attractive interaction: in the deep BEC regime one finds *λ* = 1/4 and *γ* = 0 such that *E*_*col*_(*k*) = *ħ*^2^*k*^2^/(4*m*) for large momenta. It has been demonstrated that the inclusion of collective excitations in the equation of state, as briefly outlined in the Methods and derived in refs 27 and 28, recovers the correct composite boson limit at zero temperature^28^, also providing qualitatively good results for many observable quantities across the whole crossover^27^,^29^; we follow this approach in the present work.

The superfluid (number) density *n*_*s*_ of the two-dimensional (2D) fermionic system can be written as


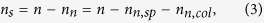


where *n* is the 2D total number density and *n*_*n*_ = *n*_*n,sp*_ + *n*_*n,col*_ is the 2D normal density due to both single-particle and collective elementary excitations^30^. For a uniform superfluid system at zero temperature *n*_*n*_ = 0 and *n*_*s*_ = *n*. As the temperature is increased the normal density *n*_*n*_ increases monotonically and, correspondingly, the superfluid density *n*_*s*_ decreases. According to Landau’s approach^30^,^31^, the two contributions to the normal density read


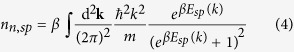


and


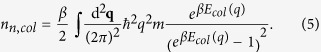


where *β* = 1/(*k*_*B*_*T*), *k*_*B*_ the Boltzmann constant and *T* the absolute temperature. The superfluid density *n*_*s*_ can also be inferred from the coefficient governing phase fluctuations in an effective action for the system^32^; it turns out that for a Gaussian-level action this approach is equivalent to setting *n*_*s*_ = *n* − *n*_*n,sp*_, ignoring the contribution from collective excitations to the superfluid density; this contribution, however, will turn out to be fundamental in the strong coupling regimes that have become recently accessible^21^.

More generally, in the extreme BCS (BEC) limit only the fermionic (bosonic) excitations contribute to the total superfluid density. As already discussed in ref. 27, the present approximation, considering the fermionic and bosonic excitations as separate, neglects the Landau damping that hybridizes the collective modes with the single-particle excitations^33^. It should be stressed, however, that the Landau damping is absent at *T* = 0, making our approximation reliable in the low-temperature limit. Moreover we also discussed^27^ that Landau damping would affect the bosonic contribution *n*_*b*_ in the BCS region, where the physics is dominated by the fermionic contribution. This interplay makes the Landau damping less relevant as far as the present work is concerned, justifying the present choice of approximation.

The effective low-energy Hamiltonian of a fermionic superfluid can be recast as that of an effective 2D XY model^34^,^35^,^36^:


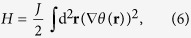


having introduced the pairing field Δ(**r**) = |Δ(**r**)|*e*^i*θ*(**r**)^ with *θ*(**r**) the so-called Nambu-Goldstone field^13^. The phase stiffness *J* is a function of the fermion-fermion attractive strength and of the temperature; it measures the energy cost associated to space variation in the phase angle *θ*(**r**) of the pairing field. Moreover the phase stiffness *J* is proportional to the superfluid number density *n*_*s*_, namely^37^


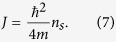


The compactness of the phase angle field *θ*(**r**) implies that 
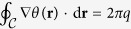
 for any closed contour 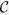. Here *q* = 0, ±1, ±2, … is the integer number associated to the corresponding quantum vortex (positive *q*) or antivortex (negative *q*). Consequently the circulation of the superfluid velocity **v**(**r**) = (ħ/m^*^)∇*θ*(**r**) is quantized according to 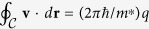 where m* = 2*m* is the mass of a Cooper pair. Formally, one can rewrite the phase angle as follows


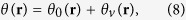


where *θ*_0_(**r**) has zero circulation (no vortices) while *θ*_*v*_(**r**) encodes the contribution of quantized vortices. Consequently, the Hamiltonian in Eq. (6) can be rewritten^37^ as *H* = *H*_0_ + *H*_*v*_ where *H*_0_ = *J*/2*∫*d^2^**r**(∇*θ*_0_(**r**))^2^ is the Hamiltonian of density oscillations, while


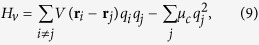


is the Hamiltonian of quantized vortices located at position **r**_*i*_ with quantum numbers *q*_*i*_, interacting through a 2D Coulomb-like potential


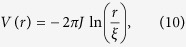


where *ξ* is healing length, i.e. the cutoff length defining the vortex core size, and *μ*_*c*_ the energy associated to the creation of a vortex^38^,^39^.

### Renormalization group analysis for a Fermi superfluid

The total number of quantized vortices varies as a function of the temperature: at zero temperature there are no vortices, however as the temperature increases vortices start to appear in vortex-antivortex pairs. Due to the logarithmic energy cost the pairs are bound at low temperature, until at the critical temperature *T*_BKT_ an unbinding transition occurs above which a proliferation of free vortices and antivortices is observed^18^. Vortex-antivortex pairs with small separation distance can screen the potential in Eq. (10) between a vortex-antivortex pair with larger distance *r*; as a consequence, the phase stiffness *J* and the vortex energy *μ*_*c*_ are renormalized^40^. In particular analyzing the effect of increasing the spatial cutoff *ξ*, thereby excluding vortex-antivortex configurations with distance smaller than *ξ*, Nelson and Kosterlitz obtained the renormalization group equations^38^,^39^,^40^


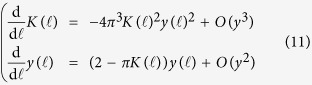


subsequently extended by Amit^41^ and Timm^42^, including next-to-leading order terms, in order to describe higher vortex densities


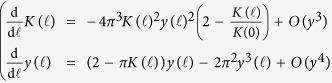


for the running variables 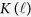 and 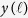, as a function of the adimensional scale 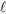 subjected to the initial conditions *K*(0) = *βJ =* *βħ*^2^*n*_*s*_/(4*m*) and *y*(0) = exp(−*βμ*_*c*_). As discussed in ref. 39, the choice of *μ*_*c*_, slightly affecting the final results, is still an open problem. The 2D XY model on a lattice with a finite difference approximation of spatial derivatives implies *μ*_*c*_ = *π*^2^*J*/2^38^. However, for the 2D XY model in the continuum it has been suggested 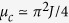 within the Ginzburg-Landau theory of superconducting films^43^,^44^,^45^ and, more recently, 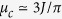 within a phenomenological BCS approximation^39^. In our study of the 2D BCS-BEC crossover with Eqs (11) we adopt *μ*_*c*_ = *π*^2^*J*/4, that is currently the most rigorous choice for superconductors and superfluids^43^,^44^,^45^. The renormalized phase rigidity *J*^(*R*)^ and the renormalized vortex energy^38^,^44^ 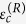 are then derived from *K*(∞) and *y*(∞). Finally, one obtains the renormalized superfluid density as


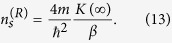


The renormalized superfluid density 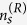 is a monotonically decreasing function of the temperature, as is the bare (unrenormalized) superfluid density *n*_*s*_; however, while *n*_*s*_ is continuous, 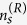 jumps discontinuously from a finite value to zero as the temperature reaches the BKT critical temperature *T*_BKT_, implicitly defined by the Kosterlitz-Nelson condition^40^:


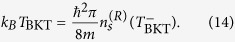


Let us verify the validity of the perturbative treatment of the renormalization group analysis. Combining Eq. (7), Eq. (14) and the definition of *μ*_*c*_ one readily sees that the expansion parameter *y* is a monotonically increasing function of the temperature, increasing from 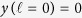 at *T* = 0, to 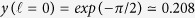 at *T* = *T*_*BKT*_. This fact suggests that even the leading-order renormalization group in Eq. (11) could give accurate results for the present problem, and in fact including the next-order correction as in Eq. (12) modifies our estimates of the critical temperature *T*_*BKT*_ by at most 1.5% over the whole crossover (see below), confirming the validity of the renormalization group analysis.

**Figure 1:**
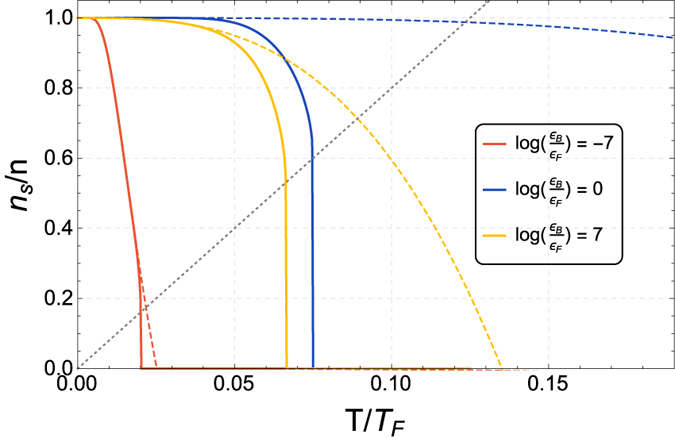
The superfluid density, for three different values of the interaction, ranging from the BCS to the BEC regime. The solid lines represent the results of the renormalization group analysis which is the central point of the present paper, whereas the dashed lines represent the unrenormalized result obtained from the single-particle and collective contributions to superfluid density, as done in ref. 27. The gray dotted line corresponds to the Nelson-Kosterlitz condition in Eq. (14), showing that the contribution from the renormalization group lowers the critical temperature. The universal jump as a consequence of the BKT appears for every value of the interaction; however the size of the universal jump and the related critical temperature are strongly interaction-dependent.

In Fig. 1 we report the renormalized and bare superfluid densities for three different values of the interacting strength, in the BCS, intermediate and BEC regimes. The renormalization of superfluid density as analyzed in Eq. (13) is more evident at higher temperatures, as the universal jump defined by Eq. (14) is approached. We also note that, although always a monotonically decreasing function of the temperature, the superfluid density exhibits different behaviors across the BCS-BEC crossover, as it can be dominated either by fermionic, single-particle excitations, in the weakly-coupled regime, or by bosonic, collective excitations, in the strongly-coupled regime.

### Phase diagram

The finite-temperature phase diagram in the present 2D case is profoundly different with respect to a three-dimensional Fermi gas as a result of the BKT mechanism just analyzed and also as a result of the MWH theorem^15^,^16^,^17^ prohibiting symmetry breaking at any finite temperature. These striking qualitative differences render a complete analysis of the 2D Fermi gas compelling both from the theoretical and experimental point of view. Let us briefly discuss the three possible phases^14^:

#### Condensation

A 2D superfluid system exhibits condensation and off-diagonal long-range order (ODLRO) only strictly at *T* = 0: this zero-temperature regime is characterized by a non-decaying phase-phase correlator 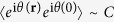, where *C* is independent of **r**, and by a finite condensate density^46^.

#### Quasi-condensation

The intermediate phase from *T* = 0^+^ to *T*_BKT_ is characterized by the phase-phase correlator showing *algebraic* quasi-long-range order 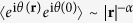 for an opportune exponent *α* > 0. Although the condensate density is strictly zero, a finite superfluid density is still present.

#### Normal state

Finally for *T* > *T*_BKT_ the system enters the normal phase, characterized by the exponential decay of the phase-phase correlator, 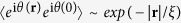 and by the absence of both superfluid and condensate.

**Figure 2:**
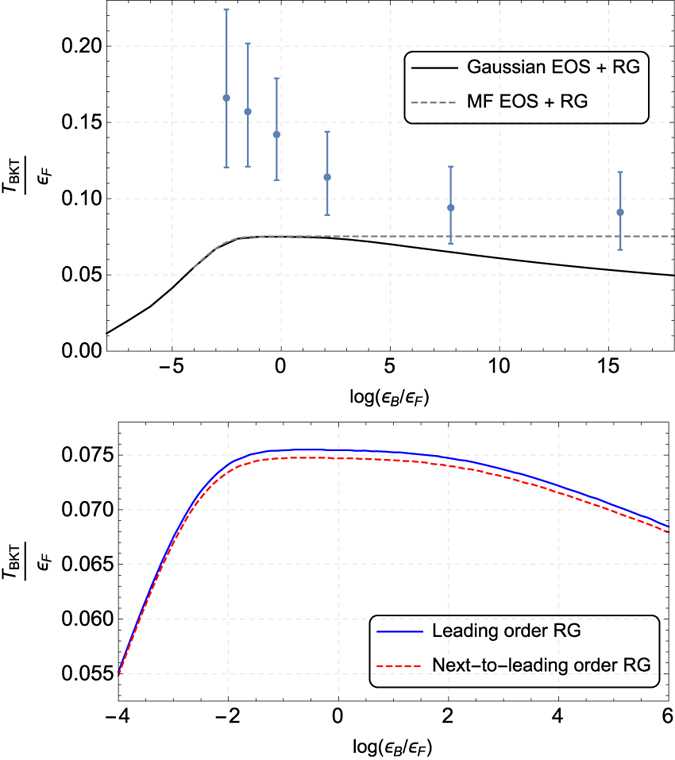
The Berezinskii-Kosterlitz-Thouless critical temperature as a function of the bound-state binding energy *ε*_*B*_. Upper panel. The dashed line is the result of renormalization group (RG) analysis, i.e. Eq. (11), of the mean-field results, whereas the solid line uses the Gaussian theory as the starting point. The blue dots represent experimental data from ref. 21. The decrease of the critical temperature in the BCS and BEC limits is due to single-particle excitations and collective excitations contributing to superfluid density, respectively. This interplay results in a higher BKT critical temperature in the intermediate regime, i.e. when 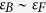. It is important to note that experimental data may be affected by systematic errors, as analyzed in the main text. Lower panel. Comparison between the Kosterlitz-Thouless renormalization group (RG) equations (11) and the next-to-leading order RG equations (12). Here, in both cases the bare superfluid density is calculated within the Gaussian theory.

The gray dashed line in Fig. 1 corresponds to the Kosterlitz-Nelson condition in Eq. (14), identifying the critical temperature *T*_BKT_, separating the normal state from the phase characterized by quasi-condensation. A determination of the critical temperature across the whole crossover is reported in the upper panel of Fig. 2, black solid line. The rapid decrease of *T*_BKT_ approaching both the BCS and the BEC limit is a consequence of the fermionic single-particle excitations and bosonic collective excitations dominating the superfluid density, respectively, rapidly decreasing the normal density as either limit is approached. A consequence of this interplay is that the critical temperature is higher in the intermediate regime (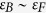), where the superfluid density is neither fermion-dominated nor boson-dominated.

The current approach, involving the inclusion of Gaussian fluctuations in the equation of state, the inclusion of bosonic collective excitations in the superfluid density along with a renormalization group analysis is able to reproduce the downward trend as the interaction get stronger; the renormalization group analysis on top of a mean-field theory would not have been sufficient to reproduce the correct trend, as shown by the gray dashed line in the upper panel of Fig. 2. In other words, as also observed elsewhere^27^,^28^,^29^, Gaussian fluctuations are required in order to correctly describe the physics of an interacting Fermi gas in the strongly-coupled limit.

The underestimation of experimental data^21^, as observed in Fig. 2 may have different causes:

- In the experiment there is a harmonic trap also in the planar direction. The effect of the trap can enhance the critical temperature with respect to the uniform system, as found in the 3D case by Perali *et al*.^47^,^48^.
- It has been argued^49^ that the algebraic decay of the first-order correlation function, presented in ref. 21 as the signature of the superfluid state, could be interpreted in terms of the strong-coupling properties of a normal-state. Experimental data in ref. 21 would then overestimate *T*_BKT_.
- The determination of the critical temperature may be affected by three-dimensional effects, the superfluid not being trapped in a strictly 2D configuration.
- On more general grounds one may argue that *T*_BKT_ > 0.125*ε*_*F*_, as experimentally observed in the BCS regime, is not compatible with the Kosterlitz-Nelson condition, signaling different mechanisms at work^27^.

For the sake of completeness, in the lower panel of Fig. 2 we plot the BKT critical temperature *T*_*BKT*_ obtained with the Kosterlitz-Thouless renormalization group equations (11) and the generalized renormalization group equations (12), starting with the bare superfluid density derived from the Gaussian theory. As previously stressed the relative difference in the determination of *T*_*BKT*_ is below 1.5% in the whole crossover. Moreover, the figure shows that this very small difference is larger in the intermediate coupling regime. (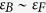).

## Discussion

In the present work we have analyzed the role of vortex proliferation in determining the finite-temperature properties of a 2D interacting Fermi gas, throughout the BCS-BEC crossover, as the fermion-fermion interaction strength is varied. Using the Kosterlitz renormalization group equations we have shown that the bare superfluid density is renormalized as the vortex-vortex potential is screened at large distances. The renormalization of superfluid density lowers the BKT critical temperature, correctly reproducing the trend observed in experimental data through a non-trivial interplay between the single-particle and collective excitations. As previously pointed out, and analyzed in ref. 49, currently available experimental data may overestimate the BKT critical temperature of the uniform system and our theoretical predictions are providing a benchmark for forthcoming experiments.

## Methods

### Equation of state

The pairing gap Δ_0_ and the chemical potential *μ* are calculated self-consistently by jointly solving the gap and number equation, as done e.g. in refs 29 and 27. The Gaussian pair fluctuations scheme^50^,^51^ has been adopted which, as opposed as the Nozières-Schmitt-Rink^52^ approach, leads to finite, converging results in 2D. The spectrum of fermionic and collective excitations, *E*_*sp*_(*k*) and *E*_*col*_(*q*) as introduced in Eqs (1) and (2), are calculated by looking at the poles of the respective Green’s functions, as analyzed e.g. in ref. 24. Accordingly, the corresponding thermodynamical grand potential has two contributions, namely the mean-field, fermionic part


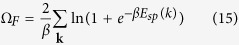


and the bosonic part


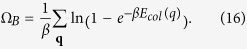


We stress that Ω_*F*_ accounts for the mean-field description of a tunable Fermi gas, whereas Ω_*B*_ includes the contribution of density waves on top of the mean-field picture.

### Data availability

The data is available upon request. Requests should be addressed to either author.

## Additional Information

**How to cite this article:** Bighin, G. and Salasnich, L. Vortices and antivortices in two-dimensional ultracold Fermi gases. *Sci. Rep.* **7**, 45702; doi: 10.1038/srep45702 (2017).

**Publisher's note:** Springer Nature remains neutral with regard to jurisdictional claims in published maps and institutional affiliations.
